# A Parsimonious Model of the Rabbit Action Potential Elucidates the Minimal Physiological Requirements for Alternans and Spiral Wave Breakup

**DOI:** 10.1371/journal.pcbi.1005087

**Published:** 2016-10-17

**Authors:** Richard A. Gray, Pras Pathmanathan

**Affiliations:** Division of Biomedical Physics, Office of Science and Engineering Laboratories, Center for Devices and Radiological Health, Food and Drug Administration, Silver Spring, Maryland, United States of America; University of Michigan, UNITED STATES

## Abstract

Elucidating the underlying mechanisms of fatal cardiac arrhythmias requires a tight integration of electrophysiological experiments, models, and theory. Existing models of transmembrane action potential (AP) are complex (resulting in over parameterization) and varied (leading to dissimilar predictions). Thus, simpler models are needed to elucidate the “minimal physiological requirements” to reproduce significant observable phenomena using as few parameters as possible. Moreover, models have been derived from experimental studies from a variety of species under a range of environmental conditions (for example, all existing rabbit AP models incorporate a formulation of the rapid sodium current, *I*_*Na*_, based on 30 year old data from chick embryo cell aggregates). Here we develop a simple “parsimonious” rabbit AP model that is mathematically identifiable (i.e., not over parameterized) by combining a novel Hodgkin-Huxley formulation of *I*_*Na*_ with a phenomenological model of repolarization similar to the voltage dependent, time-independent rectifying outward potassium current (*I*_*K*_). The model was calibrated using the following experimental data sets measured from the same species (rabbit) under physiological conditions: dynamic current-voltage (I-V) relationships during the AP upstroke; rapid recovery of AP excitability during the relative refractory period; and steady-state *I*_*Na*_ inactivation via voltage clamp. Simulations reproduced several important “emergent” phenomena including cellular alternans at rates > 250 bpm as observed in rabbit myocytes, reentrant spiral waves as observed on the surface of the rabbit heart, and spiral wave breakup. Model variants were studied which elucidated the minimal requirements for alternans and spiral wave break up, namely the kinetics of *I*_*Na*_ inactivation and the non-linear rectification of *I*_*K*_.The simplicity of the model, and the fact that its parameters have physiological meaning, make it ideal for engendering generalizable mechanistic insight and should provide a solid “building-block” to generate more detailed ionic models to represent complex rabbit electrophysiology.

## Author Summary

Understanding and preventing life-threatening irregular electrical heart rhythms includes basic experimental, numerical, and theoretical research. Computer models of the electrical dynamics of cardiac cells and impulse propagation throughout the heart are essential tools of this research. For example, simulations of electrical activity such as rotating ‘spiral waves’ have been used to understand how irregular heart rhythms are maintained. However, existing models are derived exclusively from sub-cellular data under a variety of environmental conditions and species. These models tend to be exceedingly complex including hundreds of variables and parameters which make them difficult to validate and analyze. Using experimental data from one species (rabbit) under nearly identical physiological conditions, we developed a simple model of the electrical activity of a cardiac cell derived from sub-cellular, cellular, and tissue experimental data. This model reproduces cellular excitability and its recovery as well as several important “emergent” phenomena including beat-to-beat cellular alterations and unstable spiral waves. Under some conditions, unstable spiral waves in this model give rise to continuous formation of new spiral waves (i.e., “spiral wave breakup”) which is thought to be the underlying cause of cardiac fibrillation and sudden cardiac death.

## Introduction

The simulation of action potentials (APs) and their propagation has been an integral part of the field of electrophysiology for over half a century.[1] Over a hundred cellular AP models from many regions of the heart encompassing a variety of species have been published. These “ionic” models have been derived almost exclusively from voltage clamp data from a variety of species, often recorded under conditions that are not physiological. Ionic models are increasingly complex, and are comprised of numerous sub-models (mostly transmembrane currents), and contain tens of variables and hundreds of parameters. For example, a recent meta-analysis found that 50% of the data used in the development of the ten-Tusscher-Panfilov [2] and Iyer [3] human ventricular models were of non-human origin [4]. While their complexity has many advantages, there are a variety of limitations including: over-parametrization[5]; non-uniqueness/multi-stability [6, 7]; dissimilar predictions [8–12]; limited validation outside their development domain [13] (e.g. using a cell model derived from voltage clamp data to study reentrant arrhythmias); difficulty in representing natural “physiological redundancy” (e.g. repolarization reserve [14]); interpreting the relative roles of model sub-components on model predictions; and generalizing insight from “model-specific” simulations results.

An alternative, and complementary approach compared to these ionic models, is to use “phenomenological” models which are designed to represent specific, often macroscopic phenomena (e.g., rate dependence of action potential duration)[15–19]. Phenomenological models are designed to reproduce one (or two) specific phenomena(on) very well, and are: simplistic, computationally efficient, and sometimes amenable to analytical approaches. However, unlike ionic models, phenomenological models do not provide a direct link to physiologically meaningful model parameters derived experimentally. Therefore phenomenological models provide only limited mechanistic insight, and are not amenable for reproducing numerous phenomena, nor can they be easily extended.

The two most important membrane currents for cardiac excitability are the rapid sodium current (*I*_*Na*_) and the rectifying potassium current (*I*_*K*1_), which are both highly non-linear functions of transmembrane potential (*V*_*m*_). *I*_*Na*_ is responsible for the rapid all-or-none depolarization of the AP upstroke, and *I*_*K*1_ is responsible for maintaining the resting potential near -85 mV. In fact, it has been shown recently that transfecting cells with genes encoding for cardiac *I*_*Na*_, *I*_*K*1_, and gap junctions Cx43 (only) into unexcitable somatic cells can transform monolayers into excitable tissue capable of supporting propagating “cardiac” action potentials including reentrant “spiral” waves![20]

Due to the ethical and practical limitations involved in studying human physiology, animal experiments are necessary to understand and develop clinical strategies aimed at treating the pathological mechanisms underlying heart disease. The isolated rabbit heart has been shown to share certain electrophysiological characteristics as the human, including repolarization reserve [21] and ventricular fibrillation dynamics.[22] as well as the effects of many pharmaceuticals.[23]

All existing ionic models of the rabbit ventricular AP [24–27] incorporate the *I*_*Na*_ equations from the LR1 guinea pig model,[28] albeit with different conductance values. The LR1 *I*_*Na*_ equations incorporate the gating kinetics of fast activation, fast inactivation and slow inactivation. The fast activation and fast inactivation equations were derived from voltage clamp (VC) data from spherical clusters of eleven day-old embryonic chick heart cells,[29] and slow inactivation parameters were based on VC results from sheep and pig ventricular trabeculae.[30] Obviously, there is a need to update rabbit AP models to incorporate an *I*_*Na*_ model based on rabbit data, but this is not a simple task. To date, *I*_*Na*_ activation parameters have been derived exclusively from VC protocols; however, since the *I*_*Na*_ current in the adult myocyte is too large to allow adequate voltage control under physiological conditions,[31] these VC protocols are carried out at low temperatures with low extracellular sodium concentrations or in HEK cells or oocyctes transfected with the SCN5A gene which encodes the cardiac *I*_*Na*_ channel (Na_v_ 1.5).

The first aim of this paper is to develop a “parsimonious” model for the rabbit myocyte AP based *only* on data recorded from the rabbit under physiological conditions. A model is considered parsimonious if it accomplishes a desired level of explanation or prediction with as few parameters as possible (although, as far as we are aware, there is no definitive and scientifically rigorous definition of parsimonious). For this paper, the desired level of prediction is the following well-known and important electro-physiological phenomena, measured from rabbit ventricular myocytes/tissue under nearly identical and physiological conditions: 1) steady-state inactivation as determined from voltage clamp experiments [31]; 2) action potential depolarization in single cells; 3) recovery of AP excitability in single cells[32]; and 4) action potential depolarization dynamics during propagation in the whole heart. [33] For calibration and evaluation of the model we coupled this *I*_*Na*_ model with various models of *I*_*K*1_, and also explored a range of cellular and tissue behavior in an AP model incorporating a previously published phenomenological two parameter model of repolarization.[33] The overall model will be referred to as the parsimonious rabbit (PR) model. The second aim of this paper is to demonstrate that this simple and parsimonious model is sufficient for reproducing important “emergent” complex phenomena such as alternans and spiral wave breakup, which suggests a novel physiological mechanism for arrhythmia instabilities.

## Methods

### Parsimonious Rabbit (PR) *I*_*Na*_ Sub-model Formulization

The equations of *I*_*Na*_ are of the form pioneered by Hodgkin-Huxley (HH) [1]

$$I_{Na}=g_{Na}m^{3}h(V_{m}-E_{Na}),$$ (1)

where *V*_*m*_ is the transmembrane potential, *g*_*Na*_ is the maximal conductance of *I*_*Na*_, *m* (fast activation) are gating variables, and *h* (fast inactivation), and *E*_*Na*_ is the Nernst equilibrium potential for sodium. Beeler and Reuter [34] introduced a slow inactivation gate (*j*)

$$I_{Na}=g_{Na}m^{3}hj(V_{m}-E_{Na}),$$ (2)

which has been incorporated into all of the most recent cardiac HH *I*_*Na*_ models.[24–27].

The HH equations for the gating variables are of the form:

$$\frac{dy}{dt}=\alpha_{y}(1-y)-\beta_{y}y\equiv \frac{y_{\infty }-y}{\tau_{y}},$$ (3)

where *y* represents each gating variable, *α*_*y*_(*V*_*m*_) and *β*_*y*_(*V*_*m*_) represent the on and off rate constants for gating (respectively) which are voltage dependent, *y*_∞_(*V*_*m*_) and *τ*_*y*_(*V*_*m*_) are the steady state fraction of activation or inactivation and time constant (respectively) and are functions of *V*_*m*_. Hodgkin and Huxley [1] fit their voltage clamp data to empirically determine the functions *α*_*y*_(*V*_*m*_) and *β*_*y*_(*V*_*m*_) using only 9 parameters; in contrast the LR1 *I*_*Na*_ equations contain 31 parameters. Since 1952 there has been significant advancement in the derivation of gating equations based on first principles.[35, 36] The simplest functional form for the rate equations of voltage dependent gating based on thermodynamics and chemical reaction rates are:[37]

$$\begin{array}{ccc} \alpha_{y}=\alpha_{y}^{0}\exp\left(\frac{-\delta_{y}^{}V_{m}}{k_{y}^{}}\right) & , & \beta_{y}=\beta_{y}^{0}\exp\left(\frac{\left(1-\delta_{y}\right)V_{m}}{k_{y}^{}}\right) \end{array},$$ (4)

where the constants $\alpha_{y}^{0}$, $\beta_{y}^{0}$, and 0 ≤ *δ*_*y*_ ≤ 1 are positive, while *k*_*y*_ < 0 for activation and *k*_*y*_ > 0 for inactivation. Using Eq 3 we can derive the equations for *y*_∞_(*V*_*m*_) and *τ*_*y*_(*V*_*m*_) from Eq 4:

$$y_{\infty }(V_{m})=\frac{1}{1+\exp[\frac{\left(V_{m}-E_{y}\right)}{k_{y}}]},$$ (5)

and

$$\tau_{y}(V_{m}^{})=\frac{2\tau_{y}^{0}\exp[\frac{\delta_{y}(V_{m}-E_{y})}{k_{y}}]}{1+\exp[\frac{\left(V_{m}^{}-E_{y}\right)}{k_{y}}]}.$$ (6)

There are 4 parameters per gate (*E*_*y*_,*k*_*y*_,$\tau_{y}^{0}$,*δ*_*y*_) in this formulization. The main limitation of this formulization is that *τ*_*m*_(*V*_*m*_)→0 away from *E*_*m*_ which is not realistic, because gating transformations cannot be instantaneous. For example, the LR1 equations predict *τ*_*m*_(−85*mV*) = 0.0055 *ms* and *τ*_*m*_(+30*mV*) = 0.041 *ms* at 37 C, however these values are below the resolution of voltage clamp measurements. We address this problem by removing the *V*_*m*_ dependence of *τ*_*m*_ (making it a constant, thus eliminating parameter *δ*_*m*_). To summarize, we define the novel PR *I*_*Na*_ submodel using Eqs 1 and 3, with *m*_∞_ and *h*_∞_ given by Eq 5, *τ*_*h*_ given by Eq 6, and *τ*_*m*_ equal to a constant.

### Parsimonious Rabbit (PR) *I*_*Na*_ Sub-model Calibration

All model parameters are provided in **Table 1**. Values for *E*_*h*_ and *k*_*h*_ where taken directly from Table 1 in [31]. We set *E*_*Na*_ = 65*mV*, and all other parameters were determined via manual fitting to the experimental data described below using no more than 2 significant digits; $\tau_{h}^{\max}$ and *δ*_*h*_ were computed via “inverting” Eq 6 given the two value pairs *τ*_*h*_(−80*mV*) = 4.0*ms* and *τ*_*h*_(0*mV*) = 0.45*ms*.

**Table 1:** Parameter values for “parsimonious rabbit” (PR) model.

current	parameter	value	units
*I*_*Na*_	*g*_*Na*_	11	mS/μF
	*E*_*m*_	-41	mV
	*k*_*m*_	-4.0	mV
	*τ*_*m*_	0.12	ms
	*E*_*h*_	-74.9	mV
	*k*_*h*_	4.4	mV
	$\tau_{h}^{0}$	6.80738	ms
	*δ*_*h*_	0.799163	none
*I*_*K*_	*g*_*K*_	0.3	mS/μF
	*b*	0.047	mV^-1^

Simulations required for the calibration were carried out in single cells and a one-dimensional cable by integrating the three ordinary differential equations using exact integration for the gating variables and forward Euler integration for *V*_*m*_ with dt = 0.001 ms which is actually a very conservative choice made because of the low computational cost. The fact that *τ*_*m*_ is constant provides for a well-defined minimum time constant of the model which allows for the use of larger values of dt and computational efficiency compared to other models. [38] Cable simulations were performed using a central difference approximation of the Laplacian in a 4 cm long cable using *I*_*ion*_ = *I*_*Na*_ + *I*_*K*1*L*_ (in order to establish a steady-state resting membrane potential) with diffusion coefficient (*D*) equal to 0.001 cm^2^/ms and dx = 0.01 cm, where *I*_*K*1*L*_ is the formulation of *I*_*K*1_ provided by Livshitz and Rudy [39]. *I*_*Na*_ and *V*_*m*_ signals were recorded at the center of the cable to construct the I-V curves for calibration to the experimental data as described below.

### Dynamic Current-Voltage Relationship during the Action Potential Upstroke

The current during the AP upstroke is predominately *I*_*Na*_, therefore the precise time course of *V*_*m*_(*t*) contains a wealth of information regarding *I*_*Na*_ kinetics.[40–42] In isolated myocytes the time course of *I*_*Na*_ can be estimated as $I_{ion}^{}=-C_{m}\frac{dV_{m}}{dt}$ (after the stimulus current is turned off), where *C*_*m*_ is the specific membrane capacitance which we assume is equal to 1 μF/cm^2^. During propagation, however, the total transmembrane current is *not* proportional to $\frac{dV_{m}}{dt}$; but we recently developed a methodology and justified the computation of *I*_*Na*_ as $C_{m}\left(\frac{D_{}}{{\left(CV\right)}^{2}}\frac{d^{2}V_{m}}{dt^{2}}-\frac{dV_{m}}{dt}\right)$ during the AP upstroke during planar wave propagation, where *CV* is the conduction velocity [33]. These relationships were used to calibrate our model to dynamic I-V curves from glass microelectrode *V*_*m*_ measurements during AP upstrokes from isolated rabbit ventricular myocytes[43] and from the epicardial surface during planar propagation on the surface of the isolated Langendorrff-perfused heart.[33] In isolated myocytes *V*_*m*_ traces from APs stimulated with near threshold stimuli with a latency of 2.5 ms (range of 1.2 to 3.7 ms) were chosen from a previous study[43], because this latency provided datum during the early decrease *I*_*Na*_ of near –40 mV. Similar to Joyner et al. [32] we found a slight decrease (5.2 ± 0.5 mV/ms per ms) of ${\left(\frac{dV_{m}}{dt}\right)}_{\max}$ as a function of latency which corresponds to only a 3.4% decrease compared to the traditional latency value of 1 ms.

### Recovery of Action Potential Excitability Protocol

It is well known that ${\left(\frac{dV_{m}}{dt}\right)}_{\max}$ is decreased during the relative refractory period of the AP, and this is thought to be a *cellular* phenomenon [32]. Here we assume that this phenomena (“recovery of AP excitability”) is controlled only by the voltage and time dependent *I*_*Na*_ current and the AP shape. To reproduce the experimental results of recovery of AP excitability in isolated myocytes,[32] we developed a novel “virtual protocol”. This protocol involves simulating an action potential clamp of *I*_*Na*_ using APs measured during pacing from six isolated myoctes recorded in a previous study.[43] During the 11^th^ beat the simulations were switched to current-clamp mode and stimulated with a 2 ms stimulus at various coupling intervals; the amplitude was adjusted such that the latency was 1 ms. The resulting peak *I*_*Na*_ was normalized to that recorded during the 10^th^ beat and presented as a function of coupling interval.

### Comparison with Previous Models

Since the *I*_*Na*_ equations in all previous rabbit models are identical to LR1, except for scaling (conductances), we present previous model predictions using LR1 with two conductance values (23 mS/μF and 8 mS/μF) to cover the entire range (grey lines in figures). In addition we present results from the Ebihara-Johnson (EJ) model [29] (dashed grey lines) which is identical to LR1, except it does not contain slow inactivation.

### Parsimonious Rabbit (PR) Cell Model

**Fig 1:**
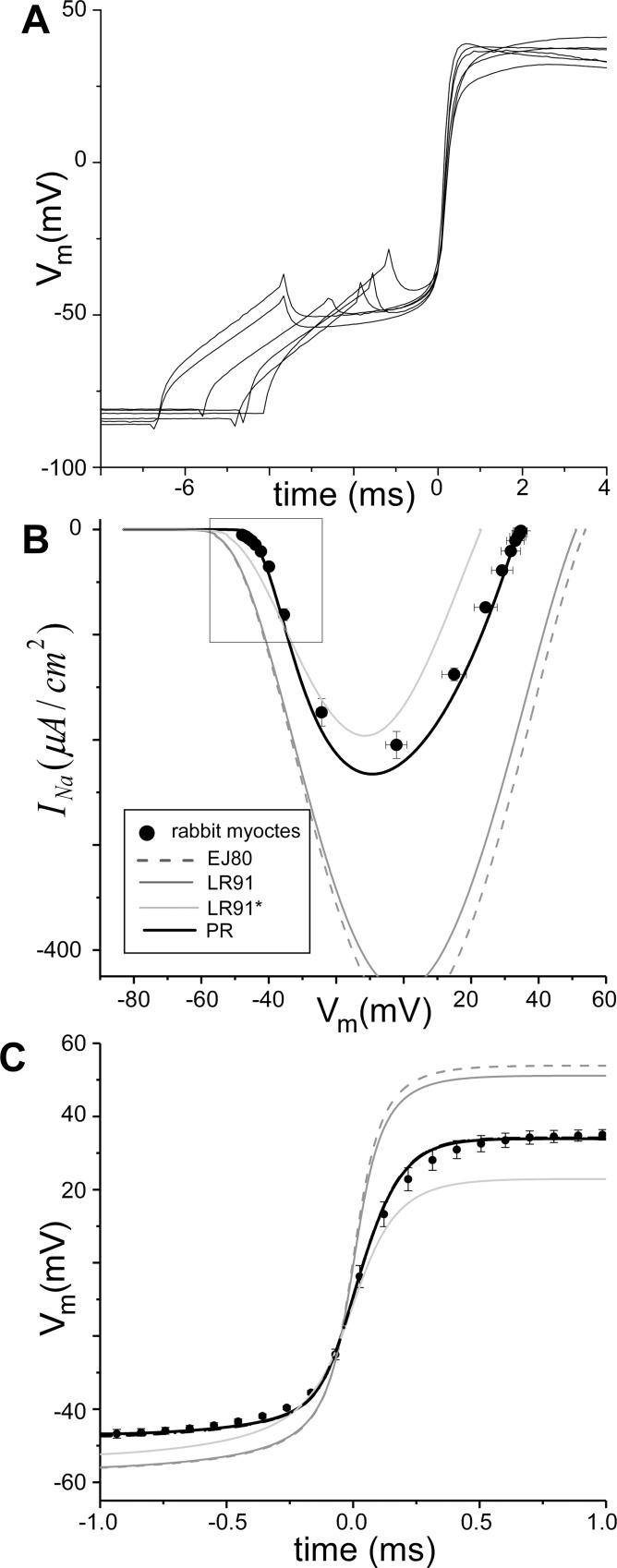
Dynamic I-V curves from isolated cells. A) Transmembrane potential (*V*_*m*_) recorded during the action potential upstroke from six isolated rabbit cells from [43]. B) Current-voltage (I-V) relationship plotted as *I*_*Na*_ (models: lines) or $-C_{m}^{}\frac{dV_{m}}{dt}$ (experiments: ●) versus *V*_*m*_. C) Predicted *V*_*m*_ signals from models (lines) and average from experiments (●) during the upstroke.

To create our parsimonious rabbit (PR) cell model, we combined our model of *I*_*Na*_ with a previously published phenomenological model of repolarization (*I*_*K*_) designed to reproduce rabbit AP repolarization during propagation:

$$I_{K}=g_{K}e^{-b\left(V_{m}-E_{K}\right)}(V_{m}-E_{K}),$$ (7)

where *g*_*K*_ is the maximal conductance of *I*_*K*_, *b* is a parameter controlling AP shape, and *E*_*K*_ is the reversal potential for potassium (set equal to the average resting potential of the six cells in **Fig 1** of -83 mV); the nominal parameters values for *I*_*K*_ are provided in **Table 1**, as previously determined.[33] We define our PR cell model via a total ionic current of *I*_*Na*_ + *I*_*K*_ in Eqs 1 and 5–7 with parameters in **Table 1**.

## Results

### Dynamic Current-Voltage Relationship during the Action Potential Upstroke

The experimental traces of *V*_*m*_ during AP depolarization of isolated rabbit ventricular myocytes are shown in **Fig 1A**; the traces are aligned to ${\left(\frac{dV_{m}}{dt}\right)}_{\max}$. The average dynamic I-V curve during the AP upstroke for these traces are plotted as symbols (● representing the mean ± standard error of the mean) in **Fig 1B**. The calibrated PR results are shown as a thick black line. Previous model predictions (grey lines) bracket the range of peak *I*_*Na*_ but do not accurately capture the initial inward current deflection of the experimental I-V relationship (see box).

**Fig 2:**
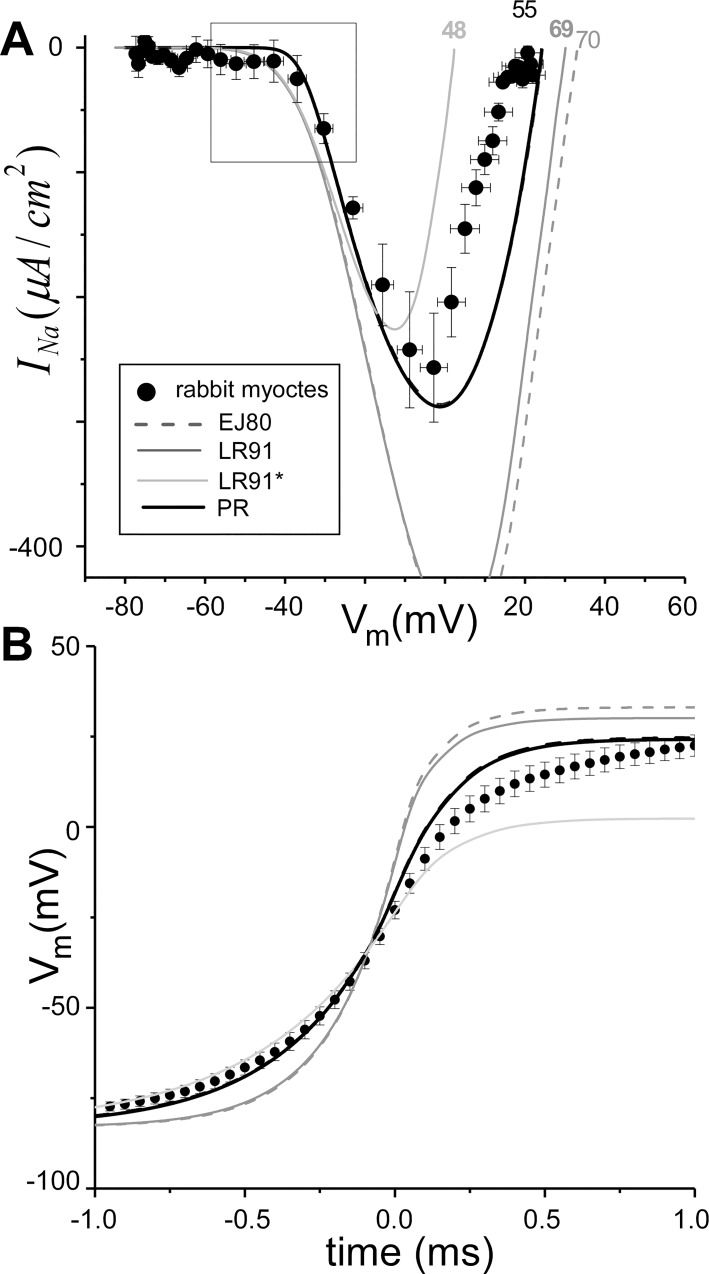
Dynamic I-V relationship during propagation. A) Current-voltage (I-V) relationship plotted as *I*_*Na*_ (models) or *I*_*ion*_ (experiments: ● from [33]) versus *V*_*m*_. Values of CV for each model are shown at the top. B) Predicted transmembrane potential (*V*_*m*_) for the action potential upstroke during propagation for the models (lines) and average from experiments (●).

The experimental dynamic I-V curves during the AP upstroke during *propagation* from [33] are shown as symbols (●) in **Fig 2A**. Similar to above, previous model predictions (grey lines) span the range of peak *I*_*Na*_ but do not accurately capture the initial inward current deflection of the experimental I-V curve (see box). Although the PR model fits the initial decrease of *I*_*Na*_ and maximum *V*_*m*_ well, it does not match the experimental dynamic I-V curve during the second half of the upstroke.

It is important to note that the experimental dynamic I-V curves for isolated myocytes (Fig 1B) is different than that obtained during propagation (Fig 2A). To quantify these differences we fit both curves to fifth order polynomials and identified the regions in which the 95% confidence intervals of these fits did not overlap (see Fig A in S1 Text). The inward current during the upstroke for myocytes was more negative compared to during propagation for -41 mV < *V*_*m*_ < -22 mV and for *V*_*m*_ > +4.5 mV.

### Recovery of Cellular Excitability

**Fig 3:**
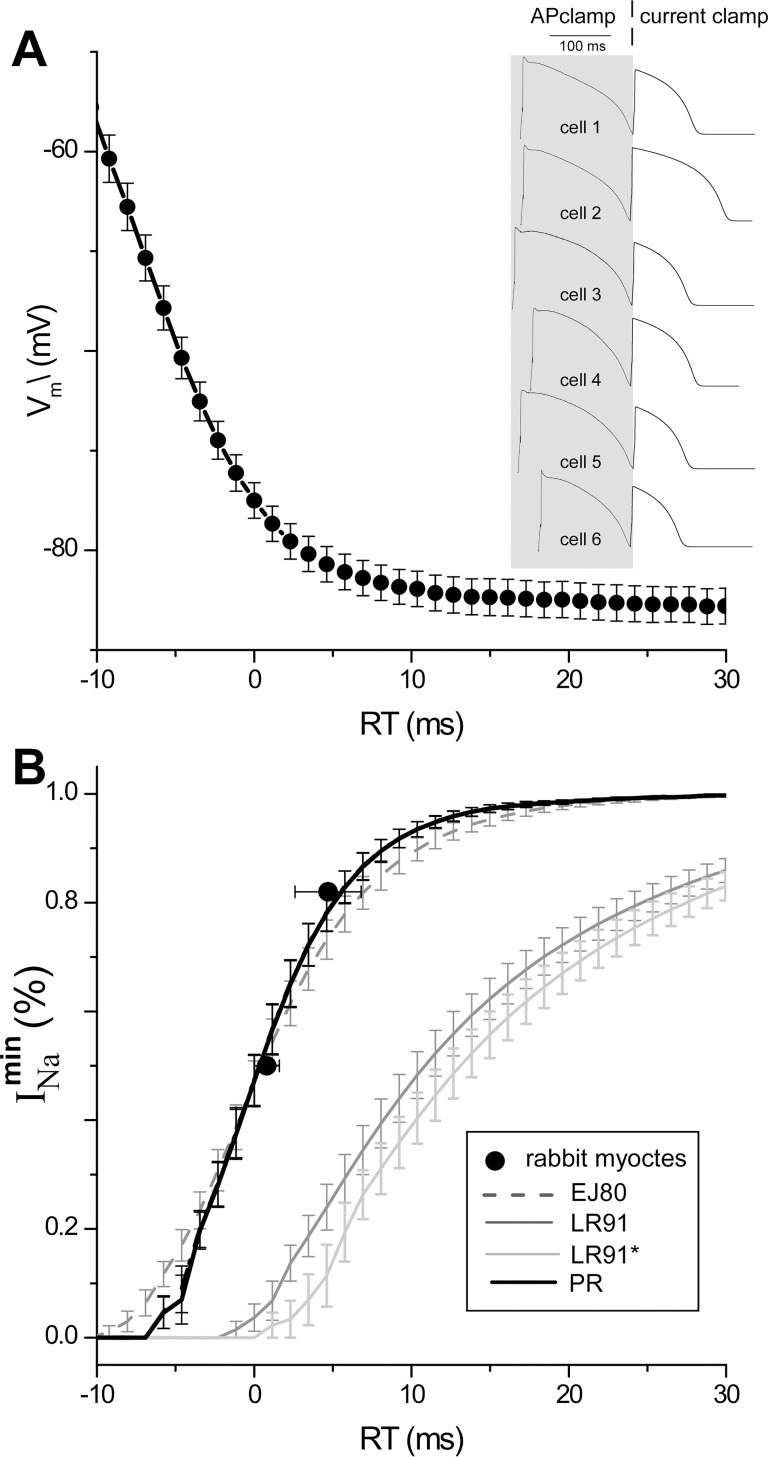
Recovery of AP excitability virtual protocol. A) Average *V*_*m*_ (●) of six experimental cells shown in inset, aligned to recovery time (RT: defined as the time at which *V*_*m*_ recovers to within 5 mV of the resting potential). B) Normalized peak *I*_*Na*_ (models: lines) or ${\left(\frac{dV_{m}}{dt}\right)}_{\max}$ (experiments: ●) as a function of RT. Experimental values of RT at 50% (0.8 ± 0.8 ms) and at 82% (4.7 ± 0.8 ms) were taken from in Table 1 of [32]. The error bars in B) reflect the variability of AP shape of the six cells (see panel A). The novel action potential clamp virtual protocol is described in the text.

The normalized magnitude of peak *I*_*Na*_ as a function of recovery time are shown in **Fig 3B**; experimental results from [32] are shown as symbols (●), PR model results are shown as a thick black line, and previous model predictions shown in grey. Simulation results (i.e., lines) include standard error bars because the virtual protocol includes six different APs and thus include the effect of normal variation of AP shape. *Unlike previous models with slow inactivation (Eq 2), the PR and EJ80 models which include only fast inactivation (Eq 1) are consistent with the experiments.*

### Steady State Activation, Inactivation, and Time Constants

**Fig 4:**
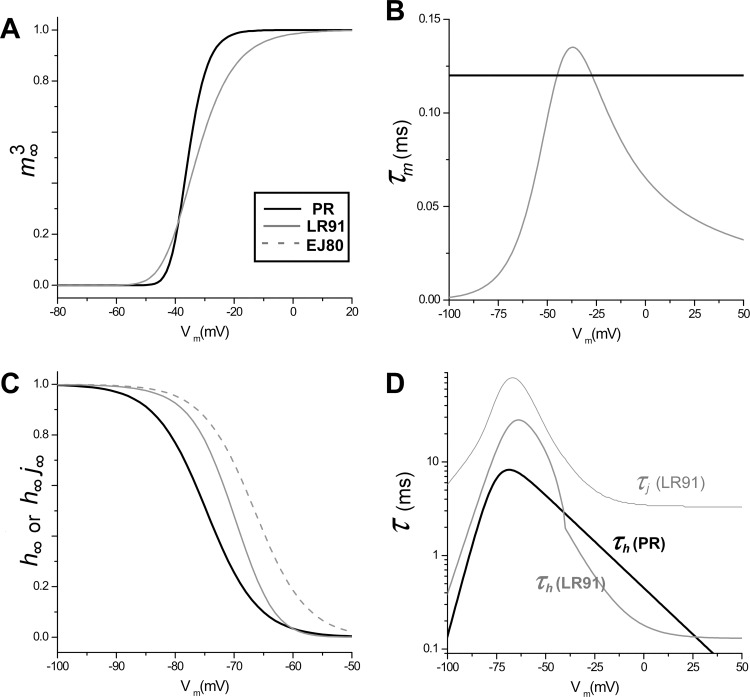
Voltage dependence of steady state *I*_*Na*_ activation (A), time constant for activation (B), steady state *I*_*Na*_ inactivation (C), and time constant for inactivation (D) for the PR (black), LR1 (grey), and EJ (dashed grey) models (note the log scale).

The voltage dependence of steady state *I*_*Na*_ activation and inactivation as well as the corresponding time constants are shown in **Fig 4** for our PR model, as well as LR1, and EJ models. Our PR model exhibits a steeper activation slope at slightly less depolarized voltages compared to LR1 and EJ (panel A). As described above, *τ*_*m*_ in the PR model does *not* exhibit voltage dependence but is similar to the mean value of LR1 and EJ (panel B). The steady state *I*_*Na*_ inactivation for PR is more negative compared to LR1 and EJ (panel C) and the maximum value of *τ*_*h*_ for PR is less than that for *τ*_*h*_ for LR1 and EJ80 and is considerably less than *τ*_*j*_ for LR1 (panel D).

### Robustness of PR *I*_*Na*_ Sub-model

The PR results in **Figs 1 and 2** represent the results from for our novel *I*_*Na*_ *sub-model only;* the results in **Fig 3** include coupling to the *I*_*K*1*L*_ sub-model. To assess the effect of repolarization current on the PR results shown in **Figs 1–3** we reran all simulations with cell models incorporating *I*_*K*1*L*_ or the phenomenological model of repolarization Eq 7 (PR cell model). The results from these simulations were nearly identical to the PR *I*_*Na*_ sub-model *only*; in fact the results are shown as thick dashed (*I*_*K*1*L*_) and dotted (*I*_*K*_) lines in **Figs 1–3** but cannot be seen because they superimpose upon the solid black line.

Simulations using our PR cell model resulted in APs with the following characteristics: $I_{Na}^{\min}=-233$μA/cm^2^, $V_{m}^{\max}=34\begin{array}{c} mV \end{array}$ and *APD* = 197*ms* for single cells; and $I_{Na}^{\min}=-289$μA/cm^2^, $V_{m}^{\max}=24\begin{array}{c} mV \end{array}$, *CV* = 55 *cm* / *s* and *APD* = 142*ms* for propagation in a cable. With the exception of *APD*, these values were not significantly altered (< 2%) compared to simulations using *I*_*K*1*L*_ [39] or *I*_*K*_ [33] indicating that the predictions of our PR *I*_*Na*_ sub-model are quite insensitive to the specific choice of repolarization current.

We performed parameter sensitivity analysis by quantifying the effect of a 1% variation of the eight *I*_*Na*_ gating parameters on eight quantities of interest; results are provided in the Table A in S1 Text as an 8x8 matrix. We also characterized the effect of AP shape on cell and tissue-level behavior for our new PR model (*I*_*Na*_ + *I*_*K*_) by varying repolarization parameters *g*_*K*_ and *b* over the entire “physiological range” for all mammals: reported values of *g*_*K*_ range from 0.1 to 0.5 mS/μF and the range of *b* values chosen, 0.03 ≤ *b* ≤ 0.05 mV^-1^, correspond to *APD*s ranging from 23 ms to 516 ms. The results are displayed in the Figs B and C in S1 Text. Over this range of *g*_*K*_ and *b*, $I_{Na}^{\min}$ varied 1.6% and $V_{m}^{\max}$ varied 1.2% in single cells, while during propagation the following quantities exhibited a fairly small dependence on *I*_*K*_ parameters: $I_{Na}^{\min}$: 1.8%; $V_{m}^{\max}$: 7.5%; *CV*: 4.5%. *The fact that action potential depolarization characteristics are fairly insensitive to repolarization in single cells and during propagation provides support for the robustness of our I*_*Na*_ *model*.

### Model Evaluation

To confirm that the data used in the model calibration is representative, we compared several model results to experimental data from previously published studies. Table B in S1 Text demonstrates that the following model results are within the range found in the literature: resting membrane potential, action potential amplitude, (*dV*_*m*_/*dt*)_max_, and *CV*.

**Fig 5:**
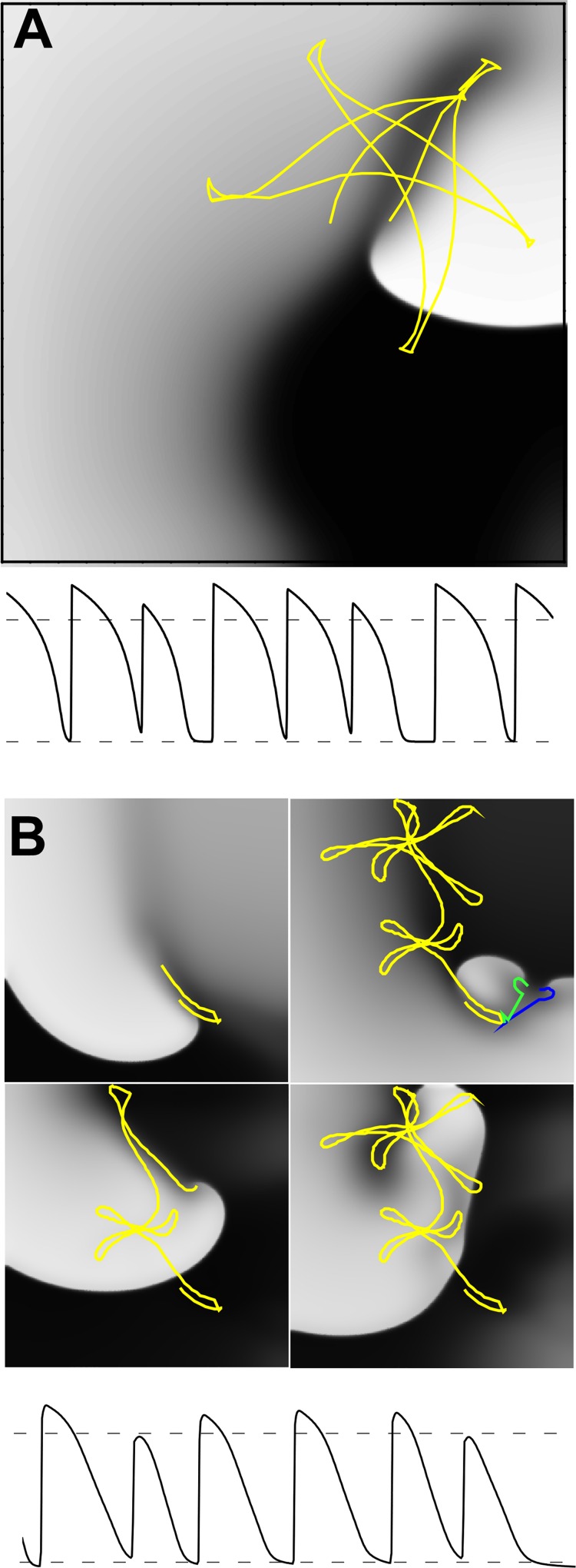
**Simulations of functional reentry for PR model (A) and Mahajan model (B).** Tip trajectories overlaid in yellow, green, and blue, 5 cm x 5 cm grids and *D* = 1 cm^2^/s.

**Fig 6:**
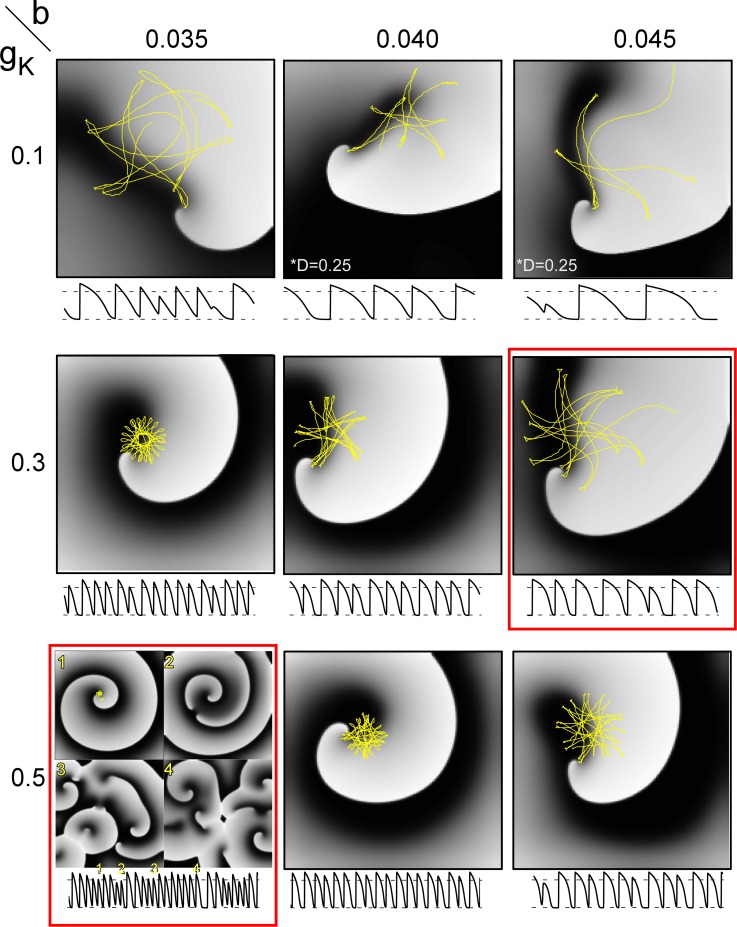
Spiral wave dynamics as a function of repolarization current *I*_*K*_ parameters (*g*_*K*_ and *b*). Snapshots of spiral wave patterns are shown in grey scale with the tip trajectories overlaid in yellow. Example *V*_*m*_ signal from one site is shown under each panel with the dashed horizontal lines indicating -83 and 0 mV. Simulations (dt = 0.02 ms and dx = 0.01 cm) were carried out on 4.8 cm x 4.8 cm grids and *D* = 1 cm^2^/s with the exception of the top two right panels where *D* = 0.25 cm^2^/s to account for the larger wavelength. Red boxes indicate that the corresponding videos are available online.

In addition, we compared the predictions of spiral waves in the PR and Mahajan (M08) models (5 cm x 5 cm) to those recorded in the whole rabbit heart by Schalij et al.[44] Schalij et al found that only fibrillation was observed in the intact heart but that, after freezing the inside of the heart, stable reentry occurred around a linear line of conduction block of ~ 2 cm with a cycle length of 130 ± 11 ms.[44] Simulation results for PR and M08 are shown in **Fig 5**. The cycle length (~ 150 ms) and line of block (~ 3.2 cm) for PR using the nominal parameter values were larger than the experiments. Incidentally, decreasing parameter *b* by only 4% decrease improved the correspondence considerably (cycle length: ~125 ms; line of block: 2.5 cm as shown in the middle, right panel of **Fig 6** below). This change is justified because the PR model does not include any repolarization kinetics and is not developed to reproduce APD shortening with rate. In contrast to the experimental results of Schalij et al., [44] the line of block in our simulations was not static, but rotated which is typical of spiral wave simulations.[2] Simulations with M08 resulted in non-sustained unstable reentry with average cycle length of length (~ 150 ms); the tip trajectory traversed the majority of the region, and eventually terminated by hitting the top boundary. There was evidence of wave break (see green and blue trajectories in **Fig 5B** consistent with previous results indicating SWB for this model in a 7.5 cm x 7.5 cm region.

The level of validation required for a model depends on its “context of use” and the consequences of incorrect model predictions[13]. Hence further validation is expected to be required for each specific context. The preliminary validation performed here is appropriate for using the model to gain insight into underlying physiological mechanisms as demonstrated below.

### Spiral Wave Dynamics

During “functional” spiral wave reentry the depolarization and repolarization processes are *inter*dependent. As shown in **Fig 6**, the dynamics of spiral waves in our model varied considerably as a function of repolarization (i.e., *g*_*K*_ and *b*). Spiral waves were not stable (i.e., rotationally symmetric) for any parameter pairs (*g*_*K*_, *b*) in the range studied. For 8 of 9 parameter pairs, the spiral wave tip followed typical non-circular trajectories, while spiral wave breakup (SWB) occurred for one parameter set (*g*_*K*_ = 0.5, *b* = 0.035). The frequency of activity for single spiral waves ranged from ~3 Hz to ~18 Hz and the frequency of activity during SWB was ~30 Hz.

### Alternans

**Fig 7:**
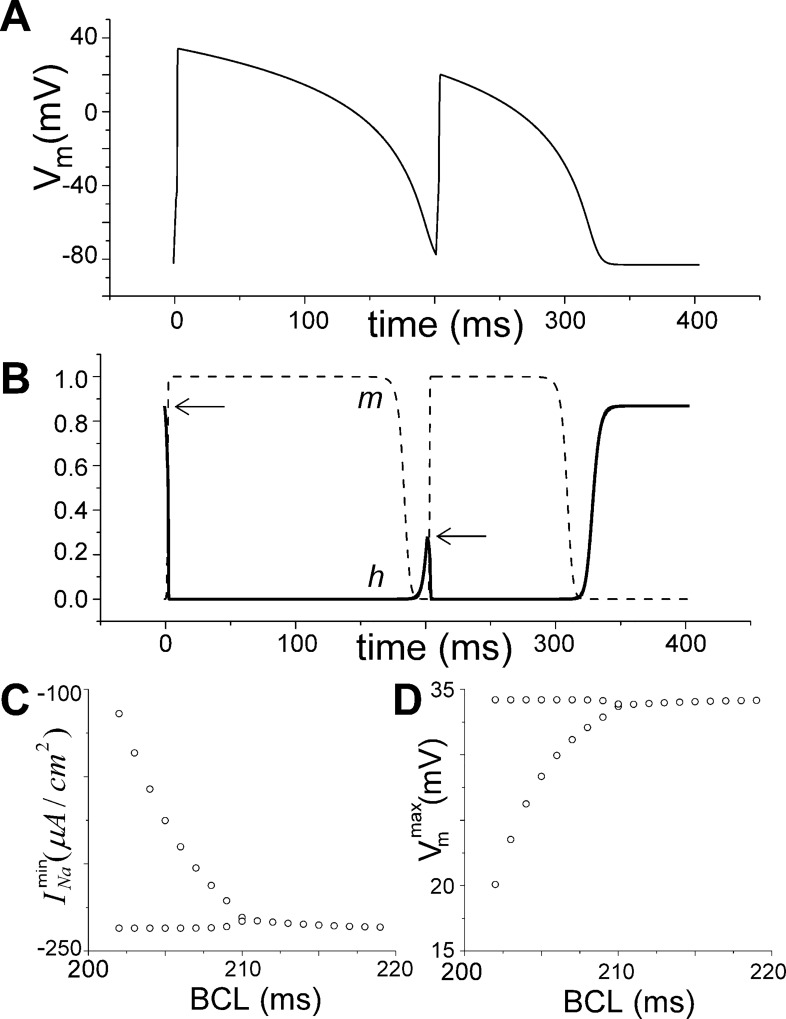
Cellular alternans in PR model during pacing. A) Beat-to-beat dynamics of sequential action potentials during periodic pacing at BCL = 202 ms. B) The corresponding dynamics of the activation *m* (dashed line) and inactivation *h* (solid line) variables. Note the similarity of *m* for subsequent beats while *h* alternates as shown by arrows. Bifurcation diagrams illustrating steady state alternans in $I_{Na}^{\min}$ C) and $V_{m}^{\max}$ D) as a function of BCL.

Since SWB was observed (albeit for parameters corresponding to *APD* = 23*ms* which is *not* consistent with normal rabbit physiology) we investigated whether cellular alternans occurred in our PR model. We paced the PR cell model with a 2 ms stimuli of -20 pA/pF and found that alternans occurred for cycle lengths between 202 and 210 ms, as shown in **Fig 7**, which is within the range (190–240 ms) reported previously for isolated rabbit myocytes.[26]. Since the PR model contains no repolarization kinetics, we hypothesized that *APD* alternans occurred as a result of alternations of peak *I*_*Na*_, which resulted in alternans in $V_{m}^{\max}$, which caused alternans in *APD*. Since *I*_*Na*_ is negligible except during depolarization, *APD* is a function of only $V_{m}^{\max}$ (and can be computed by integrating Eq 7 with initial condition $V_{m}^{}=V_{m}^{\max}$). Since alternations in the variable *h* were more pronounced than *m* (see **Fig 7B**), we hypothesized that only *I*_*Na*_ *in*activation kinetics are necessary for cellular alternans. A “reduced” two variable (*V*_*m*_,*h*) model, in which variable *m*^3^ was replaced with function $m_{\infty }^{3}(V_{m}^{})$ and *g*_*Na*_ was reduced to 5.8 mS/μF (to maintain *CV* = 55*cm* / *s*), confirmed that *I*_*Na*_ *in*activation by itself was capable of generating cellular alternans in our PR model.

## Discussion

Traditionally, cardiac AP models have been developed from equations and parameters derived from voltage clamp experiments. Many currents have voltage-dependent gating variables whose characterization via voltage clamp provides intuitive and meaningful parameters. However, in order to obtain such parameters it is necessary to isolate individual currents either chemically, functionally, or genetically. In addition, the relationship of voltage-clamp derived parameters to AP characteristics in single cells and during propagation is complex, non-intuitive, and often involves the interaction of multiple variables.

Here we provide an alternative approach in which we calibrate our model in such a way as to reproduce certain AP behavior under physiological conditions *while including all available voltage clamp data*. In this way, our PR model is both physiologically meaningful and parsimonious. It is physiological because it incorporates HH-like equations and parameters with only *one phenomenological* parameter *b* which controls AP shape. It is parsimonious because it was constructed was constructed to reproduce the specific set of experimental data sets (Figs 1–3) that capture important electrophysiological behavior with as few parameters as possible. In addition, we have recently shown that this model is mathematically identifiable (i.e., not over parameterized)[45]. Incidentally, as described by Biktashev et al. [46] FitzHugh–Nagumo models fail to reproduce some features of cardiac ionic models such as: slow repolarization, slow sub-threshold response, non-Tikhonov asymptotic properties of excitability [47], wave front dissipation [48], and different action potential amplitude in single cells versus propagation [9] which our model captures (see Fig 2).

Physiological models are developed using a set of experimental calibration data, which for our model is for rabbit tissue and myocytes at physiological temperature and heart rate. Our approach should be relatively straight-forward to implement for other species, although this will require the high-fidelity measurement of the dynamic I-V curve during propagation [33] and in single cells as well as the recovery of excitability in isolated myocytes [32]. As far as we are aware, our PR model is the first to include dynamic I-V curves during propagation into its development (i.e., calibration) which we believe is superior to simply adjusting *g*_*Na*_ to match CV. We believe that proper extensions of our PR model will include additional constraints obtained using appropriate data sets during recalibration. One reason we think our model is useful is because it has only one inward current which is non-zero only during depolarization, and only one outward current. Unlike traditional models in which multiple combinations of currents can give rise to the same AP shape [49], in our model repolarization is characterized only by a time-independent, voltage dependent current (*I*_*K*_), and therefore, *I*_*K*_ parameters completely determines AP shape [33]. In addition, each current typically incorporates one or two variables (often gates). Despite the non-uniqueness of the relationship between model parameters and AP shape, model developers have largely ignored the interaction of the various currents and variables (components) in simulations. For a model with *n* > 1 components, the total number of possible interactions between two or more components, excluding the full model of all *n* components, is 2^*n*^ − *n* − 2. Traditional models have at least 10 components, which results in over 1000 interactions! Characterizing these interactions appropriately both physiologically and mathematically continues to be a significant challenge.

[2, 31, 50, 51]The simplicity of our model allows unique *physiological* insight regarding the interaction of depolarization and repolarization processes. We found that *I*_*K*_ conductance (*g*_*K*_) is related to the cycle length of reentry (see Fig 6) consistent with the experimental findings of Warren et al. in guinea pig [52]. In addition, our PR model exhibited the expected relationship between *APD* and spiral wave behavior [53]; specifically, the frequency of activity for single spiral was inversely related to *APD*, and also inversely related to the region covered by the spiral wave tip.

Surprisingly, our PR model (comprised of only a HH *I*_*Na*_ current with only two gating variables and a time-independent, voltage-dependent *I*_*K*_ current) exhibited important “emergent” behavior including cellular alternans (Fig 7), unstable spiral waves (Figs 5 and 6), and even spiral wave breakup (Fig 6, bottom left panel). We initially thought that alternans and spiral wave breakup would require dynamic repolarization variables with time constants with values close to the *APD* capable of generating *APD* alternans. We only discovered spiral breakup serendipitously while performing a sensitivity analysis of the spiral wave meandering behavior on the *I*_*K*_ repolarization parameters. It is important to note, however, that the parameter set for which breakup occurred (*g*_*K*_ = 0.5,*b* = 0.035) resulted in a rate (~30 Hz) which is twice that observed in experiments.[54, 55]

While our PR model reproduces important physiologically meaningful behavior, it does have limitations. It should be noted that the alternans resulting from the interaction of *I*_*Na*_ and *I*_*K*_ are the ‘minimal’ requirements, other currents such as *I*_*to*_ and *I*_*CaL*_ may either enhance or suppress this behavior. Our PR model does not include intracellular calcium dynamics, nor the majority of repolarization membrane currents. It should be noted that the Tyrode’s solution used to perfuse the hearts from which the propagation I-V experimental data were obtained (Fig 2) in the presence of 15 mM diacetyl monoxime, however the longitudinal propagation velocity was 59 ± 3 cm/s which is within the range reported for the rabbit without any uncoupling agents (see Table B in S1 Text). The *I*_*Na*_ equations are of HH type, Markov models are required to replicate certain features of voltage clamp experiments including drug binding kinetics.[50] Importantly, the *APD* in our model is dependent on $V_{m}^{\max}$ which depends on the stimulus current, which is different in single cells compared to in tissue during propagation. When the PR model is stimulated to unphysiologically large *V*_*m*_ the *APD* is unphysiologically long; to avoid this in tissue simulations we initiated propagation in the cable by holding *V*_*m*_ at the end (1 mm) at 0 mV for 2 ms.

One significant difference between our *I*_*Na*_ sub-model and previous rabbit *I*_*Na*_ sub-models is that we did not include a slow inactivation gate. While slow inactivation of *I*_*Na*_ can be justified from voltage clamp experiments [31], we have found that it can induce unphysiological post repolarization refractoriness as shown in Fig 3 and [51]. In addition, the *I*_*Na*_ sub-model in the existing rabbit models are the same as LR1 in which the slow inactivation kinetics were derived from sheep and calf trabeculae muscles in which spatial homogeneity of *V*_*m*_ and VC control are lacking. We believe that much more research is needed to appropriately incorporate slow inactivation of *I*_*Na*_ (as well as late *I*_*Na*_) into AP models, especially those used to simulate propagation. Incidentally, ten Tusscher et al. interpolated experimental time constants of fast and slow activation as a function of voltage over the range -80 to -40 mV in which data is not available to values more than four times values outside of this range (see Fig 1 D & E in [2]).

Our novel PR model provides unique and physiological generalizable insight into the relationship among gating kinetics and AP behavior in single cells and during propagation. We show unequivocally that only two currents (*I*_*Na*_,*I*_*K*_) and three variables (*V*_*m*_,*m*,*h*) are required to reproduce cellular alternans and spiral wave breakup. In fact, only two variables (*V*_*m*_,*h*) are required to produce alternans and implicate a destabilizing effect resulting from the interaction of the non-linear rectification of *I*_*K*_ [56] and the voltage and time dependence of *h*. We believe that our PR model is an ideal tool to quantify excitability and propagation related to a variety of important physiological and pathophysiological issues. For example our model can be used to study the effects of drugs (our model predicts that an 83% decrease of *g*_*Na*_ is required for propagation failure), genetic mutations of SCN5A such as LQT3, and the differences between atrial and ventricular tissue. In addition we believe that it provides a solid “building-block” to develop more realistic rabbit AP models by including additional sub-models (e.g., *I*_*CaL*_, *I*_*to*_, *I*_*Kr*_ and intracellular calcium handling).

All experimental data and PR simulation results presented in this paper (with the exception of the videos) will be made available online at Research Gate. The model will be uploaded to CellML.

## Supporting Information

S1 Text Supplementary information including Tables A and B as well as Figs A, B and C.
(DOCX)

S1 Video Spiral wave dynamics *g*_*K*_ = 0.3*mS* / *μF* and *b* = 0.0.45*mV*^−1^. (AVI)

S2 Video Spiral wave dynamics *g*_*K*_ = 0.5*mS* / *μF* and *b* = 0.0.35*mV*^−1^. (AVI)
