# Supplementary material for: A Parsimonious Model of the Rabbit Action Potential Elucidates the Minimal Physiological Requirements for Alternans and Spiral Wave Breakup
Source: PLoS Comput Biol. 2016 Oct 17;12(10):e1005087. doi: 10.1371/journal.pcbi.1005087 (PMC5066986; doi:10.1371/journal.pcbi.1005087)
Supplement: S1 Text — Supplementary information including Tables A and B as well as Figs A, B and C. (DOCX) [file pcbi.1005087.s001.docx]

**A Parsimonious Model of the Rabbit Action Potential Elucidates the Minimal Physiological Requirements for Alternans and Spiral Wave Breakup**

by Richard A. Gray & Pras Pathmanathan

*PLoS Computational Biology* (PCOMPBIOL-D-16-00631R2)

**SUPPLEMENTARY MATERIAL**

1. Cell versus tissue (propagation) dynamic I-V curves
2. Parameter sensitivity analysis
3. Comparison with previous experimental results
4. Movie legends
5. Cell versus tissue (propagation) dynamic I-V curves

We fit the dynamic I-V curves of single cells (Fig. 1B) and from the whole heart during propagation to fifth order polynomials with R^2^ = 0.99 and R^2^ = 0.97, respectively. These fits are shown in **Figure A** (myocyte: solid line; tissue; dashed line) along with their 95% confidence intervals (corresponding thin lines).

**Figure A**

1. Parameter sensitivity analysis for “Parsimonious Rabbit “(PR) model

We performed two parameter sensitivity analyses. First we quantified the effect of varying eight$I_{Na}$ parameters on eight quantities of interest (QOIs) as shown as an 8x8 matrix in **Table A**. Second, we also characterized the effect of repolarization parameters $g_{K}$ and $b$ on certain cell and tissue-level behavior by running simulations while varying them to cover the entire “physiological range” for all mammals (see **Figures B & C**).

We choose the following eight QOIs (2 cellular, 0D, and 6 corresponding to propagation, 1D): $I_{Na}^{\min}(0D)$, $V_{m}^{\max}(0D)$, $I_{Na}^{\min}(1D)$, $V_{m}^{\max}(1D)$, $CV$, $I_{C}^{\max}(1D)$, $I_{m}^{dur}(1D)$, and $RE_{50}$, where $CV$ is conduction velocity, $I_{C}^{\max}(1D)=\left(C_{m}\frac{\partial V_{m}}{\partial t}\right)_{\max}^{1D}$, $I_{m}^{dur}(1D)$ is the time interval between the maximum and minima of $I_{m}(1D)$, and $RE_{50}$ is % recovery of $I_{Na}^{\min}(0D)$at RT=0.8 ms[1]. Specifically, we adjusted each parameter individually by ±0.5% and computed the corresponding % change in each QOI; the corresponding 8x8 matrix is provided in **Table A**. Here we list the combinations that had an “amplifying” effect (i.e, > 1% change in QOI): $I_{Na}^{\min}(0D)$: $E_{m}(+1.1)$, $E_{h}(-2.9)$,$\delta_{h}(+2.4)$; $V_{m}^{\max}(0D)$: $E_{h}(-3.1)$, $k_{h}(+2.1)$, $\delta_{h}(+8.6)$; $I_{Na}^{\min}(1D)$:$E_{h}(-1.8)$, $\delta_{h}(+2.4)$; $V_{m}^{\max}(1D)$: $E_{h}(-4.1)$, $k_{h}(+3.0)$,$\delta_{h}(+12.5)$; $CV$: $E_{m}(+1.1)$ , $\delta_{h}(+1.1)$; $I_{C}^{\max}(1D)$: $E_{m}(+1.1)$, $E_{h}(-2.4)$, $\delta_{h}(+2.5)$; $RE_{50}$: $E_{h}(-1.4)$, $\delta_{h}(+2.5)$. $I_{Na}$ parameters$E_{h}$ and $\delta_{h}$had the largest effect on model predictions; recall that $E_{h}$ influences the voltage dependence of the inactivation time constant (see Eqn, (6)); the % change in 7/8 QOI were smaller when$E_{h}$ was modified in $h_{\infty}$ (Eqn. (5)) but not in $\tau_{h}$ (Eqn. (6)) as seen in **Table A**. This highlights the important role of the *voltage dependence* of $\tau_{h}$ (not so much its scaling). It should be noted that in many cases the representation of$\tau_{h}\left(V_{m}\right)$ in HH models is extrapolated an order of magnitude near $E_{h}$ where data is especially sparse (see Fig. 1D in Ref #[2]; Fig. S1E. in Ref[3]).

Table S1: Parameter sensitivity matrix for PMR model

| **QOI ->**  **parameter** | $I_{Na}^{\min}(0D)$ | $V_{m}^{\max}(0D)$ | $I_{Na}^{\min}(1D)$ | $V_{m}^{\max}(1D)$ | $CV$ | $I_{C}^{\max}(1D)$ | $I_{m}^{dur}(1D)$ | $RE_{50}$ |
| --- | --- | --- | --- | --- | --- | --- | --- | --- |
| $g_{Na}$ | 0.663 | 0.379 | 0.585 | 0.558 | 0.287 | 0.661 | -0.355 | 0.108 |
| $E_{m}$ | **1.106** | 0.352 | 0.523 | 0.600 | 0.870 | **1.055** | -0.709 | 0.233 |
| $k_{m}$ | 0.057 | -0.016 | 0.036 | 0.016 | 0.020 | 0.081 | -0.177 | 0.050 |
| $\tau_{m}$ | -0.444 | -0.100 | -0.524 | -0.272 | -0.321 | -0.693 | 0.532 | 0.086 |
| $E_{h}$ | **-2.944** | **-3.010** | **-1.823** | **-4.131** | **-1.018** | **-2.36** | 0.887 | **-6.706** |
| $k_{h}$ | 0.451 | **2.086** | 0.070 | **3.023** | 0.207 | 0.486 | 0.00 | 0.240 |
| $\tau_{h}^{0}$ | 0.413 | 0.651 | 0.146 | 0.840 | 0.109 | 0.254 | -0.177 | -0.340 |
| $\delta_{h}$ | **2.377** | **8.627** | 0.817 | **12.537** | **1.08** | **2.539** | -0.354 | 0.639 |

% change in QOI for a 1% change in parameter

We considered the two reversal potentials ($E_{Na}=+65mV$ and$E_{K}=-83mV$) as “environmental parameters” because they are determined by the temperature and ion concentrations inside and outside the cell, and did not include them in the above analysis.

We also characterized the effect of repolarization parameters $g_{K}$ and $b$ on certain cell and tissue-level behavior for our new PR model by running simulations while varying them over the entire “physiological range” for all mammals: reported values of $g_{K}$ range from 0.1 to 0.5 mS/μF and the range of $b$values chosen, 0.03 $\leq b\leq$ 0.05 mV^-1^ as shown in **Figure B**. Over this range, the values of action potential duration ($APD$) vary between 23 and 516 ms (**Figure C**) and $I_{C}^{\min}$ranges from to -6.1 to -0.74 μA/cm^2^ (data not shown), while the depolarization properties change very little (**Fig.B**). Hence, this choice of $g_{K}$ and $b$ encompasses a large range of repolarization behavior. Example action potentials are shown in **Fig. C**. We performed this analysis to determine if coupling our $I_{Na}$ionic model to our phenomenological $I_{K}$ model would adversely affect the ability of the cell model to reproduce the cellular depolarization process that the $I_{Na}$model was designed to reproduce.

**Figure B**

**Figure C**

1. Comparison with previous experimental results

Table B. Selected Experimental Results: Adult New Zealand rabbit (temperature: 36±1 C)

| **QOI** | **Unit** | **PR**  **value** | **values from literature: mean**± **SEM (BCL) [citation #]** |
| --- | --- | --- | --- |
| myocyte (M) |  |  | Microelectrode recordings |
| $RMP^{M}$ | $mV$ | -83 | -84.6 ± 0.5 [4]; -82.8 ± 0.7 [5]; -82.7 ± 0.4 [6]; -82.4 ± 0.6 [7]; -81.4 ± 1.3 [8]; -81.3 ± 0.2 [9]; -81 ± 2 [10]; -78.8 ± 2 [11] |
| $APA^{M}$ | $mV$ | 117 | 114 ± 1 [7]; 117 ± 4 [4]; 121 ± 3 [11]; }; 122 ± 8 [8];  127 ± 1 [6]; 127 ± 3 [10] |
| $\left(dV_{m}/dt\right)_{\max}^{M}$ | $mV/ms$ | 233 | 113 ± 8 [7]; 146 ± 18 [4]; 309 ± 11 [5]; 395 ± 21 [6] |
| tissue (T) |  |  | floating microelectrode and extracellular mapping (CV) |
| $RMP^{T}$ | $mV$ | -83 | -85 ± 1 [12]; -83 ± 1 [13]; -82 ± 1 [14];  -81.7 ± 0.6 [15]; -81 ± 3 [16] |
| $APA^{T}$ | $mV$ | 107 | -95 ± 1 [13]; 106 ± 2 [16]; 118 ± 2 mV [12]; 120 ± 2 [14] |
| $CV_{L}$ | $cm/s$ | 55 | 50 ± 6 (300) [17]; 55 ± 0.3 (250) [18];  61 ± 2 (350) [19]; 67 ± 4 (250) [15] |

* NOT comprehensive

RMP: resting membrane potential

APA: action potential amplitude

CV: conduction velocity

1. Movie legends

**Gray_Fig6_gk3_b045_2d.avi**. Spiral wave dynamics $g_{K}=0.3mS/\mu F$ and $b=0.0.45mV^{-1}$.

**Gray_Fig6_gk5_b035_2d.avi**. Spiral wave dynamics $g_{K}=0.5mS/\mu F$ and $b=0.0.35mV^{-1}$.

**References**

1. Joyner RW, Ramza BM, Osaka T, Tan RC. Cellular mechanisms of delayed recovery of excitability in ventricular tissue. Am J Physiol. 1991;260(1 Pt 2):H225-H33.

2. ten Tusscher KH, Noble D, Noble PJ, Panfilov AV. A model for human ventricular tissue. Am J Physiol Heart Circ Physiol. 2004;286(4):H1573-H89. doi: 10.1152/ajpheart.00794.2003 [doi];00794.2003 [pii].

3. Grandi E, Pasqualini FS, Bers DM. A novel computational model of the human ventricular action potential and Ca transient. Journal of Molecular and Cellular Cardiology. 2010;48(1):112-21. PubMed PMID: WOS:000273883600015.

4. de Groot JR, Veenstra T, Verkerk AO, Wilders R, Smits JP, Wilms-Schopman FJ, et al. Conduction slowing by the gap junctional uncoupler carbenoxolone. Cardiovasc Res. 2003;60(2):288-97. PubMed PMID: 14613858.

5. Belardinelli L, Liu G, Smith-Maxwell C, Wang WQ, El-Bizri N, Hirakawa R, et al. A novel, potent, and selective inhibitor of cardiac late sodium current suppresses experimental arrhythmias. J Pharmacol Exp Ther. 2013;344(1):23-32. doi: 10.1124/jpet.112.198887. PubMed PMID: 23010360.

6. Golod DA, Kumar R, Joyner RW. Determinants of action potential initiation in isolated rabbit atrial and ventricular myocytes. Am J Physiol. 1998;274(6 Pt 2):H1902-H13.

7. McIntosh MA, Cobbe SM, Kane KA, Rankin AC. Action potential prolongation and potassium currents in left-ventricular myocytes isolated from hypertrophied rabbit hearts. J Mol Cell Cardiol. 1998;30(1):43-53. PubMed PMID: 9500863.

8. Xiao GS, Zhou JJ, Wang GY, Cao CM, Li GR, Wong TM. In vitro electrophysiologic effects of morphine in rabbit ventricular myocytes. Anesthesiology. 2005;103(2):280-6. PubMed PMID: 16052110.

9. Fedida D, Giles WR. Regional variations in action potentials and transient outward current in myocytes isolated from rabbit left ventricle. J Physiol. 1991;442:191-209. PubMed PMID: 1665856; PubMed Central PMCID: PMC1179885.

10. Puglisi JL, Yuan W, Bassani JW, Bers DM. Ca(2+) influx through Ca(2+) channels in rabbit ventricular myocytes during action potential clamp: influence of temperature. Circ Res. 1999;85(6):e7-e16. PubMed PMID: 10488061.

11. Bassani RA, Altamirano J, Puglisi JL, Bers DM. Action potential duration determines sarcoplasmic reticulum Ca2+ reloading in mammalian ventricular myocytes. J Physiol. 2004;559(Pt 2):593-609. doi: 10.1113/jphysiol.2004.067959. PubMed PMID: 15243136; PubMed Central PMCID: PMC1665117.

12. Gluais P, Bastide M, Caron J, Adamantidis M. Risperidone prolongs cardiac action potential through reduction of K+ currents in rabbit myocytes. Eur J Pharmacol. 2002;444(3):123-32. PubMed PMID: 12063072.

13. Biagetti MO, Quinteiro RA. Gender differences in electrical remodeling and susceptibility to ventricular arrhythmias in rabbits with left ventricular hypertrophy. Heart Rhythm. 2006;3(7):832-9. doi: S1547-5271(06)01240-9;10.1016/j.hrthm.2006.03.010.

14. Fedorov VV, Lozinsky IT, Sosunov EA, Anyukhovsky EP, Rosen MR, Balke CW, et al. Application of blebbistatin as an excitation-contraction uncoupler for electrophysiologic study of rat and rabbit hearts. Heart Rhythm. 2007;4(5):619-26. doi: 10.1016/j.hrthm.2006.12.047. PubMed PMID: 17467631.

15. Wiegerinck RF, Verkerk AO, Belterman CN, van Veen TA, Baartscheer A, Opthof T, et al. Larger cell size in rabbits with heart failure increases myocardial conduction velocity and QRS duration. Circulation. 2006;113(6):806-13. doi: 10.1161/CIRCULATIONAHA.105.565804. PubMed PMID: 16461816.

16. Valverde ER, Biagetti MO, Bertran GR, Arini PD, Bidoggia H, Quinteiro RA. Developmental changes of cardiac repolarization in rabbits: implications for the role of sex hormones. Cardiovasc Res. 2003;57(3):625-31. doi: S0008636302007915.

17. Knisley SB, Hill BC. Effects of bipolar point and line stimulation in anisotropic rabbit epicardium: assessment of the critical radius of curvature for longitudinal block. IEEE Trans Biomed Eng. 1995;42(10):957-66.

18. Thakor NV, Ranjan R, Rajasekhar S, Mower MM. Effect of varying pacing waveform shapes on propagation and hemodynamics in the rabbit heart. Am J Cardiol. 1997;79(6A):36-43. PubMed PMID: 9080865.

19. Schalij MJ, Lammers WJ, Rensma PL, Allessie MA. Anisotropic conduction and reentry in perfused epicardium of rabbit left ventricle. Am J Physiol. 1992;263(5 Pt 2):H1466-H78.
